# Post-Hospital Syndrome: A New Challenge in Cardiovascular Practice

**DOI:** 10.5935/abc.20150141

**Published:** 2015-11

**Authors:** Evandro Tinoco Mesquita, Larissa Nascimento Cruz, Bruna Melo Mariano, Antonio José Lagoeiro Jorge

**Affiliations:** Universidade Federal Fluminense, Niterói, RJ – Brazil

**Keywords:** Patient Readmission / trends, Stress, Physiological, Patient Discharge, Comorbidity / trends

## Abstract

The image of the hospital representing the modern medicine and its diagnostic and therapeutic advances becomes more evident in the face of an aging population and patients with multiple comorbidities requiring highly complex care. However, recent studies have shown a growing number of hospital readmissions within 30 days after discharge. The post-hospital syndrome is a new clinical entity associated with multiple vulnerabilities that contribute to hospital readmissions. During hospitalization, the patient is exposed to different stressors of physical, environmental, and psychosocial natures that trigger pathophysiological and multisystemic responses and increase the risk of complications after hospital discharge. Patients with a cardiac disease have high rates of readmission within 30 days. Therefore, it is important for cardiologists to recognize the post-hospital syndrome since it may impact their daily practice. This review aims at discussing the current scientific evidence regarding predictors and stressors involved in the post-hospital syndrome and the measures that are currently being taken to minimize their effects.

## Introduction

The organizational model of hospital assistance originated in Sri Lanka and in the Arab world with the emergence of wards, specialized care, and establishment of the role of the physician in patients’ care. This model of health care arrived in Europe with the Crusades and became established initially in monasteries, but gradually dissociated from the spiritual influence. Over the past centuries, hospitals consolidated their roles as centers for professional training, specialized care, and development of new technologies. Part of the success of contemporary medicine is attributed to the hospitals, reflected by the increased survival of seriously ill patients and improvement in surgical techniques, culminating with a reduction in morbidity and mortality^1^.

Hospitals also have substantial administrative challenges. Peter Drucker, the greatest thinker in the field of administration of the 20th century, recognized the hospital as the company with the greatest complexity and management challenges. In a hospital, human resources, processes, and technologies promote health care outcomes that impact the patients and the costs associated with their care^2^.

The emergence of modern cardiology occurred in the hospital environment with the development of new technologies - oscilloscopes/cardiac monitors, defibrillators, and coronary angiography. These technologies opened the way to the establishment of coronary care units, interventional cardiology, and cardiac surgery. Cardiology professionals have in a modern hospital a place to assist acute conditions, perform procedures of high complexity, and train and educate new professionals. Hospitals specialized in cardiology and functioning as tertiary and quaternary centers are nuclei of cardiovascular care where patients seek solutions for complex cases.

**Figure 1:**
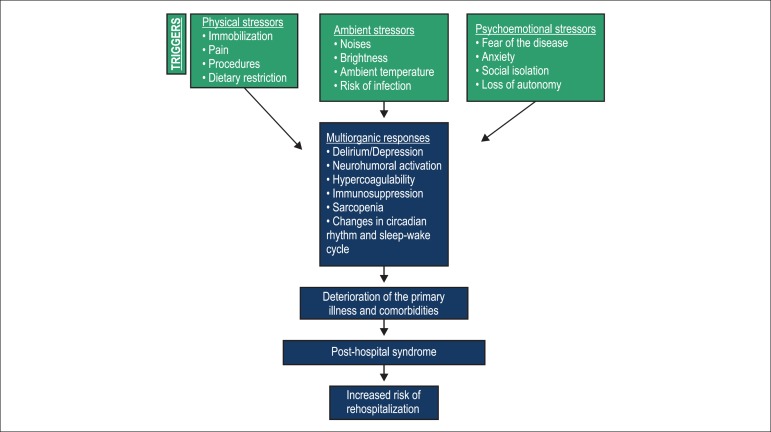
Representative model of the etiopathogenesis of the post-hospital syndrome and hospital readmission.

The association of the high complexity of the patients with the aging of the population and the presence of multiple comorbidities increase the occurrence of stressors during the period of hospitalization^3-5^. These factors are predictors of the phenomenon of rehospitalization, which consists in hospital readmission within 30 days after a patient is discharged from the hospital, due or not to the underlying pathology that led to the first hospitalization. Rehospitalization would be, therefore, one of the causes of the post-hospital syndrome (Figure 1).

The aim of this study is to discuss the current scientific evidence regarding predictors and stressors involved in the post-hospital syndrome and measures currently taken to minimize its effects.

### The post-hospital syndrome

The hospital as an important, dynamic, adaptive, and complex center is a recent and progressively consolidating paradigm. A group at Yale University led by cardiologist Harlan M. Krumholz has coined the term *hospitalomics*. This transdisciplinary group has innovatively developed important perspectives about different factors that impact patient care, producing robust scientific knowledge about the effectiveness of the hospitals in the United States (US) and the variability in health care outcomes and costs^6^. In 2013, Krumholz described a new clinical entity called post-hospital syndrome^7^. This new syndrome is a result of different stressors imposed on the patients during hospital stay and rendering them vulnerable. As a consequence, multiple complications with profound impact after discharge emerge and lead to readmissions and additional cost burden to the health system^8^.

The mechanisms involved in the syndrome induce organic abnormalities such as hypercatabolism, immunosuppression, hypercoagulability, and increased sympathetic activity. These mechanisms may be associated with different types of stressors during hospital stay or be a result of the underlying disease. These stressors, which include constant alarm noises, low temperatures, and excess brightness, modify the circadian rhythm and sleep quality of the patient. Multiple blood drawings for complementary tests causing pain and discomfort are imposed as well on the patient, who also endures physical, emotional, mental, and spiritual distress. These factors result in a higher rate of cardiovascular and cerebrovascular events and further aggravate eventual cognitive and motor deficits, contributing to the emergence of multiple vulnerabilities during hospital stay^6,7^.

Elderly and very elderly (above 80 years) patients, in particular, are the most vulnerable to the post-hospital syndrome. Therefore, upon arrival of these patients to the hospital, we must dedicate attention to their risk of developing delirium, malnutrition (due to prolonged fasting), hypovolemia (due to dehydration), deterioration of sarcopenia (from immobility), and social isolation^6,7^.

### Rehospitalization and cardiovascular disease

Cardiovascular diseases are the third most common cause of hospitalization covered by the Brazilian Unified Health System. Acute myocardial infarction (AMI), heart failure (HF), and stroke are the most prevalent clinical conditions among cardiocirculatory disorders in Brazil and in the US.

Data obtained from 2.6 million hospital admissions of Medicare beneficiaries have recently shown a high rate of rehospitalization within 30 days in the US^3^. This has a direct impact on health care costs in the country, and has resulted in changes in the calculation of a hospital’s readmissions payment adjustment factor under the Hospital Readmission Reduction Program.

Hospital readmissions, particularly during the first 30 days after discharge, may be associated with a natural progression of the patient's baseline cardiac disease, deterioration of previous comorbidities, emergence of a new clinical or surgical condition, or even polypharmacy increasing the risk of adverse events^4,5^. Rehospitalization is associated with allostatic stressors promoting psychoemotional changes during a patient's hospitalization^6^.

During the hospital stay, most patients with AMI receive dual antiplatelet therapy and undergo vascular procedures, remaining for approximately 48 to 72 hours in a coronary care unit. In the absence of complications, they are discharged within 4 to 6 days^7^. In the US, these patients have a high rate of readmission within 30 days (one in each five AMI patients returns to the hospital), promoting additional burden to the health system^7,8^. Cardiologists have already recognized that vascular complications, gastrointestinal tract bleeding, and hypotension due to vasodilators may cause readmission of patients with AMI and recurrence of coronary ischemic phenomena^6^.

Among patients with HF, the approximate rate of readmission within 30 days of discharge is 24%. Readmissions worsen a patient’s prognosis^9^. Patients with HF are usually older, have multiple comorbidities, use several medications, and are seen by different doctors. With that, the participation of a multidisciplinary team, education of the patient and the family, and detailed care plan during hospital-home transition becomes critical^10^. Half of all readmissions of patients with HF are considered to be due to associated cardiovascular causes. Also, no significant differences in rehospitalization rates are seen among the different HF phenotypes^11^.

Some physiological characteristics may be used to evaluate if an HF patient is at risk for readmission: jugular venous pressure, levels of cardiac biomarkers (B-natriuretic peptide [BNP]), markers of neurohumoral activation, and clinical signs of congestion^11,12^. Possible deteriorations in renal function and comorbidities not associated with heart disease, such as diabetes mellitus, obesity, and chronic obstructive pulmonary disease (COPD)^11^, should be taken into account during hospitalization. The risk of readmission also increases as a result of psychosocial and socioeconomic factors promoting poor treatment adherence, and scarce monitoring after discharge^13^.

Starting this year, payments for readmissions within the first 30 days for some clinical conditions considered preventable, including HF, will be reevaluated by the Hospital Readmission Reduction Program^13^. Hospitals are likely to have a profound financial impact from this decision, which has motivated important research on the phenomenon of rehospitalization and development of strategies for its prevention. Among these strategies are interventions during and after hospital discharge, such as a discharge plan, telemonitoring, home visits, and home care programs^14^.

Hospital to Home, an initiative of the American College of Cardiology, presents different strategies to reduce readmission rates, and offers an opportunity to exchange experiences emphasizing the importance of patient-centered care and education, and education of family members and caregivers involved in the patient's recovery. In parallel, the post-hospital syndrome begins to be identified as a new window of opportunities to reduce rehospitalization^10^.

### Measures to reduce the impact of the post-hospital syndrome and rehospitalization

Among patients admitted for treatment of HF, pneumonia, or COPD who are readmitted within 30 days, the cause of readmission is not necessarily the same as that for the initial admission^9^. Only 37%, 29%, and 36% of the patients with these disorders, respectively, are readmitted for the same cause that led to the initial admission. In addition to HF, pneumonia, and COPD, other causes of readmission include gastrointestinal infection, mental illness, metabolic disorders, and trauma. An important feature of this syndrome is that patients more likely to present an event leading to readmission within 30 days cannot be identified by the severity of the illness that led to their initial hospitalization^15^.

Health professionals focus their approach on the acute illness that led to the hospital admission. This approach fails to take into account stressors that may have triggered the primary cause for the admission, such as metabolic, physiological, and psychoemotional problems. Therefore, it is important to identify the stressors that during hospitalization contribute to the multiple vulnerabilities and possible triggers that may lead to rehospitalization. These stressors include sleep changes modifying the circadian rhythm, physical inactivity, pain, anxiety/depression, social isolation, noise, multiple blood drawings, loss of autonomy, dietary changes, and modifications in ambient brightness and temperature. The theories of how these stressors modify physiological responses are beginning to be elucidated. Recent studies using polysomnography have observed changes in sleep pattern during hospitalization and correlated these changes with possible neurohumoral, prothrombotic, and inflammatory abnormalities that increase the risk of cardiovascular events after hospitalization. Prolonged fasting and malnutrition during hospitalization are known to change the metabolic and immune systems and induce loss of muscle strength and increased risk of fall.

**Figure 2:**
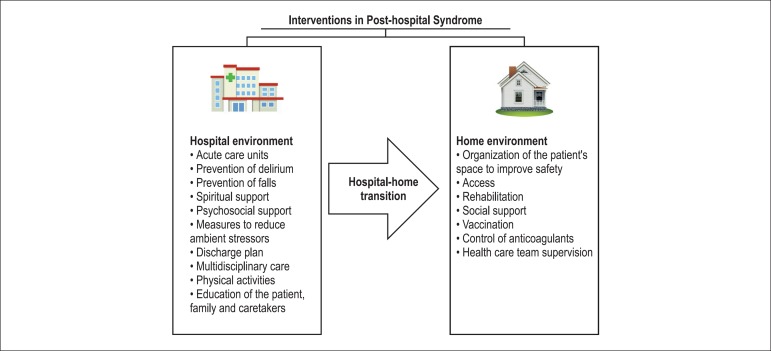
Prophylactic interventions in post-hospital syndrome.

Another important aspect that reduces the risk of the syndrome is the correct medication reconciliation at hospital discharge. Patients often interrupt medications upon leaving the hospital because they believe that the only medications they should take at home are those used before the hospital admission. It is fundamental that a pharmacist along with the physician develop a list of the medications in a clear and accurate manner to ensure that both the patients and their relatives understand the correct use of these medications^16^. It is also necessary to clearly inform the patients and their families and caregivers how to recognize the signs of adverse events associated with the drugs and which tests and physiological parameters should be carried out to ensure the effectiveness of the treatment and to minimize its adverse effects (Figure 2).

Cardiologists, hospitalists, emergency physicians, and intensivists, along with the multidisciplinary team and hospital managers, should recognize this new syndrome and seek to mitigate known factors that lead to preventable readmissions. Together, they all contribute to the sustainability of the health sector and reduce the suffering of the patients and their families. This topic deserves currently not only a transdisciplinary perspective, but also operational research at our institutions in partnership with academic centers so we can understand the magnitude and the mechanisms involved in the problem, and identify scientific solutions based on evidence to mitigate the different elements involved in this syndrome.

Some hospitals have developed care units specially designed to minimize the stressors identified by Krumholz. In the case of hospitalization of elderly patients, the initiatives have been based on a study published in 1995 by Landfeld. Since then, the units of acute care for the elderly managed by geriatricians and with a multidisciplinary approach have been able to reduce the costs and time of hospitalization, while maintaining the functional status of elderly patients^17^.

## Conclusion

Cardiologists today have an important role in leading actions to continuously improve assistance care and identify strategies to promote patient safety and reduce the impact of the hospitalization, including the recognition of the post-hospital syndrome. The study of the effects of hospital stressors and hospital-home transition measures also contributes to reduce potential sources of waste that currently impact the public and private health care systems throughout the world.
